# Recent Advances in *Astragalus membranaceus* Anti-Diabetic Research: Pharmacological Effects of Its Phytochemical Constituents

**DOI:** 10.1155/2013/654643

**Published:** 2013-11-17

**Authors:** Kojo Agyemang, Lifeng Han, Erwei Liu, Yi Zhang, Tao Wang, Xiumei Gao

**Affiliations:** ^1^Tianjin State Key Laboratory of Modern Chinese Medicine, 312 Anshanxi Road, Nankai District, Tianjin 300193, China; ^2^Noguchi Memorial Institute for Medical Research, P.O. Box LG 581, Legon, Accra, Ghana

## Abstract

The disease burden of diabetes mellitus is increasing throughout the world. The need for more potent drugs to complement the present anti-diabetic drugs has become an imperative. *Astragalus membranaceus*, a key component of most Chinese herbal anti-diabetic formulas, has been an important prospect for lead anti-diabetic compounds. It has been progressively studied for its anti-diabetic properties. Ethnopharmacological studies have established its potential to alleviate diabetes mellitus. Recent studies have sought to relate its chemical constituents to types 1 and 2 diabetes mellitus. Its total polysaccharides, saponins, and flavonoids fractions and several isolated compounds have been the most studied. The total polysaccharides fraction demonstrated activity to both types 1 and 2 diabetes mellitus. This paper discusses the anti-diabetic effects and pharmacological action of the chemical constituents in relation to types 1 and 2 diabetes mellitus.

## 1. Introduction

Diabetes mellitus (DM) has been reported as an epidemic and an increasing disease burden throughout the world [1, 2]. It is a chronic disease characterized by high blood glucose levels resulting from defects in insulin production and action. Types 1 and 2 are the most prevalent. Type 1 is characterized by lack of insulin production caused by autoimmune destruction of pancreatic beta cells. Type 2 results from the ineffective use of insulin due to insulin resistance and deficient glucose metabolism [1, 3, 4]. Research for novel anti-diabetic drugs to complement those in present clinical use has intensified over the years.

Plant medicine has been important in present anti-diabetic drug research. The prospects of a number of medicinal plants, herbal formulations, and natural products with anti-diabetic effects have been reported [5–7]. Notable among such is *Astragalus membranaceus* (AM). It is a Fabaceae flowering plant recorded in various pharmacopoeias as a herbal immunomodulator and an anti-diabetic drug. Its roots have been used in many state-approved Chinese herbal formulas for the treatment of diabetes [6, 7]. A recent publication by Wei et al. (2011) [7] identified it as the most frequently prescribed herbal medicine for diabetes treatment in China. Several ethnopharmacological studies have established its pharmacological significance [6, 8]. Recent studies have progressively sought to identify the lead compounds involved in inducing its anti-diabetic effects. Its polysaccharides, saponins, and flavonoids fractions and a number of single isolated compounds have been studied. The pharmacological processes and mechanism of action of these constituents have also been studied [9–11]. This paper considers it as an important anti-diabetic drug prospect. Thus, we review advances in its anti-diabetic research with emphasis on the pharmacological prospects of its chemical constituents in relation to types 1 and 2 DM. The following database systems were considered for data collection: PubMed, SpringerLink, Wiley Online Library, Science Direct, and China National Knowledge Infrastructure (CNKI)-China Academic Journal Network Publishing Database (CAJD).

## 2. Ethnopharmacology Effects of AM on Diabetes Mellitus 

The roots of AM have a long history for the treatment of diabetes-related symptoms in China. In traditional Chinese medicine, it is used to reinforce Qi in order to induce urination, consolidate the exterior, express toxins outward, and make new tissues grow [7, 12]. A number of studies have emphasized its pharmacological relation to diabetes mellitus. Earlier ethnopharmacological studies analyzed various crude extracts for their anti-diabetic activities and their possible pharmacological processes. They were studied as a single extract or as part of a compound formula and were reported to have demonstrated potentials of attenuating DM and their associated complications. They were generally observed to have lowered increasing blood glucose and lipid levels, improved insulin sensitivity, and also corrected several pathological indicators of DM and its complications [6, 13–16]. In a clinical study of the effect of AM on insulin sensitivity, AM decoction reduced fasting blood glucose and homeostatic model assessment (HOMA) levels in type 2 DM patients [14]. Anti-diabetic studies on *Qilan Tangzhining* capsule, a Chinese herbal anti-diabetic formula containing AM, showed its potential to reduce blood glucose levels and improve lipid profiles in streptotozocin-induced diabetic rats [16]. A number of pharmacological processes for inducing these anti-diabetic effects have been suggested. Some of which include the suppression of macrophage- and cytokine-induced inflammatory responses, stimulation of insulin signal transduction, and lowering of the hyperglycemic effects of glucagon in experimental animals. Its mechanism of action has been associated with several enzymes, proteins, and molecular markers such as peroxisome-proliferator-activated receptor gamma (PPAR*γ*), phosphatidylinositide-3-kinase (PI-3-K), and Na^+^ K^+^-ATPase, among others [10, 14, 16–18]. Further studies have sought to elucidate the phytochemical constituents inducing these anti-diabetic effects. 

## 3. Phytochemical Constituents 

Several classes of organic compounds, namely, *Astragalus* polysaccharides, saponins, flavonoids, isoflavonoids, sterols, amino acids, and volatile oils, have been isolated from AM. The polysaccharides, saponins, and flavonoids are the major chemical constituents demonstrating biological activity to DM [19, 20].

### 3.1. Polysaccharides

The polysaccharides of AM are by extraction methods water-soluble and -insoluble glucans and heteropolysaccharides. Astragalans I, II, and III are polysaccharides extracted by hot water. Astragalan *Ι* was elucidated as a neutral heteropolysaccharide containing *D*-glucose, *D*-galactose, and *L*-arabinose in the ratio of 1.75 : 1.63 : 1. It has a molecular weight of 36 kD. Astragalans *ΙΙ* and *ΙΙ* *Ι* were *α*-(1,4)-glucans with molecular weights of 12 kD and 34 kD, respectively [21, 22]. APS I and APS II were isolated by water extraction and alcohol precipitation technique. Structural and content analyses showed that APS I consisted of arabinose and glucose in the ratio of 1 : 3.45. ASP II consisted of rhamnose, arabinose, and glucose in the ratio of 1 : 6.25 : 17.86 [22, 23]. Acidic polysaccharides such as AMem-P, AH-1 and APSID3 have also been isolated [24–27]. AMem-P is a complex acidic polysaccharide with a molecular weight of 60 kD. It consists mainly of hexuronic acid and has terminal and *α*-1,5-linked-arabinofuranose, terminal and *β*-1,3-, *β*-1,4-, *β*-1,6-linked, 3,6-branched-*D-*galactose, and 2,4-branched-*L*-rhamnose residue groups attached [24]. Other *Astragalus* polysaccharides include AH-2, AE, AEF-1, and AEF-2, and astroglucans A, B, and C [27–29].

### 3.2. Saponins

The saponin content of AM consists mainly of triterpene saponins. Structurally, they are cycloartane triterpene glycosides with one-to-three sugars attached at the 3-, 6-, and 25-positions. Kitagawa (1983) reported the isolation of several cycloartane triterpenoids such as astragaloside I–VIII [30–32] and isoastragalosides I and II [30]. Astragalosides VII and VIII were elucidated as saponins with oleanane skeleton [32]. Azukisaponin V methyl ester has been isolated and identified as an oleanane-type triterpene saponin [33]. An astragaloside malonate has also been identified as malonylastragaloside [34]. Several other astragalus saponins including isoastragalosides III and IV, astramembrannin II, cyclogaleginoside B, cycloaraloside A, brachyoside B, cyclocanthoside E, cyclounifolioside B [27, 35, 36], and astramembranosides A and B [33] have also been isolated. 

### 3.3. Flavonoids

Flavonoids of varying structures have been isolated from AM. They are mainly in structural groups of flavones, isoflavones, isoflavanones, and pterocarpans. Kaempferol, isorhamnetin, rhamnocitrin, kumatakenin and rhamnocitrin-3-glucoside and quercetin-3-glucoside have been isolated as flavones [27]. Formononetin, ononin, calycosin, calycosin-7-*O*-*β*-*D*-glucoside-6′′-*O*-malonate, 3′-methoxy-5′-hydroxy-isoflavone-7-*O*-*β*-*D*-glucoside, and (3*R*)-2′,3′-dihydroxy-4′,7-dimethoxyisoflavone have been isolated as isoflavones [27, 37, 38]. The isoflavanones include 2′-hydroxy-3′,4′-dimethoxyisoflavone-7-*O*-*β*-*D*-glucopyranoside, 2′-hydroxy-3′,4′,7-trimethoxyisoflavone, 2′,7-dihydroxy-3′,4′,7-trimethoxyisoflavone, 3′,4′-dimethoxyisoflavone-7-*O*-*β*-*D*-glucoside, 8,2′-dihydroxy-4′,7-dimethoxyisoflavone, and 2′,3′,7-trihydroxy-4′-methoxyisoflavone [27]. The reported pterocarpans include 3,9,10-trimethoxypterocarpan, (6a*R*,11a*R*)-10-hydroxy-3,9-dimethoxypterocarpan, and 9,10-dimethoxypterocarpan-7-*O*-*β*-*D-*glucopyranoside [27, 38, 39]. 

## 4. Pharmacological Effects of *Astragalus* Chemical Constituents on Diabetes Mellitus

**Figure 1:**
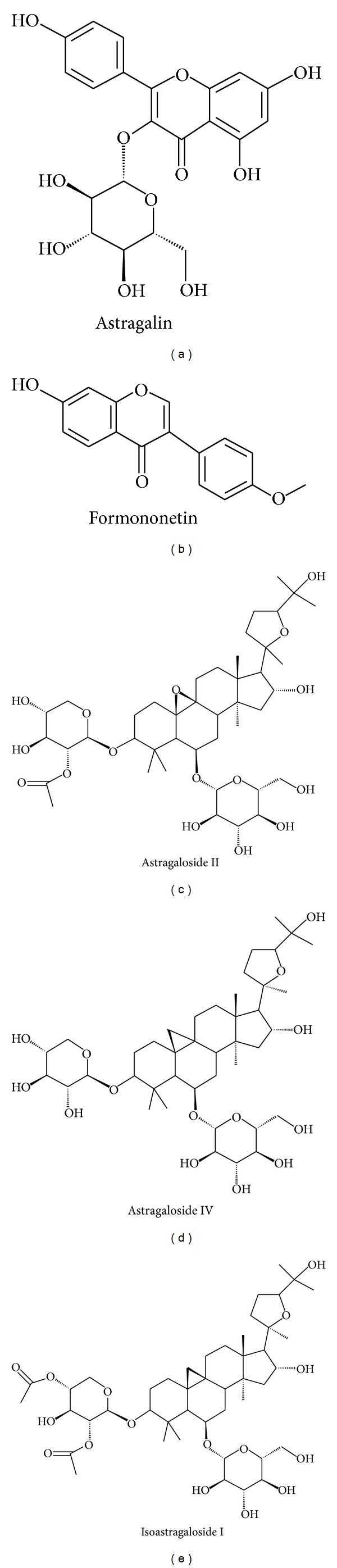
Phytocompounds of *Astragalus membranaceus* demonstrating anti-diabetic effects.

The polysaccharides (APS), saponins (ASS), and flavonoids (ASF) fractions of AM have been the most studied for their anti-diabetic effects on types 1 and 2 DM. Several single isolated compounds including astragalin, formononetin, astragalosides I, II, and IV, and isoastragaloside I (Figure 1) have also been analyzed. Their pharmacological processes and mechanism of action on types 1 and 2 DM have been reported. 

### 4.1. Type 1 Diabetes Mellitus

Type 1 DM is caused by autoimmune destruction of pancreatic beta cells. The polysaccharides fraction (APS) has been the only constituent demonstrating activity to type 1 DM. It lowered the incidence rate and postponed the onset of type 1 DM in nonobese type 1 diabetes mellitus (NOD) mice [40–42]. It also attenuated autoimmunal insulitis, increased the proliferation of pancreatic beta cells, and decreased apoptotic beta cell mass [43–45]. APS was postulated to have induced immunoprotective effects in type 1 diabetic NOD models. This potential has been widely investigated. Chen et al. (2001) and others evaluated the immunomodulatory effect of APS on CD4^+^ and CD8^+^ T cells. APS was observed to have decreased lymphocytic inflammation of pancreatic islets in type 1 noobese diabetic (NOD) mice. It was also reported to have lowered the proliferation of CD4^+^  and CD8^+^  T cells [41, 42, 46]. The CD4^+^  and CD8^+^  T cells have been implicated in inflammatory response, apoptosis, and autoimmunity leading to type 1 DM [47, 48]. APS may protect pancreatic beta cells from autoimmune destruction through the regulation of inflammatory and apoptotic responses. 

#### 4.1.1. Immunomodulation of Inflammatory Response

 The anti-inflammatory effect of APS was studied mainly on the secretory cytokines of CD4^+^ T helper cells. Naive CD4^+^ T cells differentiate into T helper cells 1 (Th1) and 2 (Th2) for inflammatory response and autoimmunity. The Th1 expresses secretory cytokines such as interferon gamma (IFN*γ*), tumor necrosis factor-alpha (TNF-*α*), interleukin-2 (IL-2), and IL-I*β* that induce inflammation and intracellular autoimmune responses. The Th2 is noted for IL-4, IL-5, IL-10,and IL-13 production for extracellular immunity and counteraction of Th1 inflammatory response [49, 50]. APS has demonstrated the potential to lower the expression of Th1 cells and regulate Th1 and Th2 imbalance in *in vivo *diabetic models. Chen and Yu (2004) in molecular immunomodulatory studies reported a possible correction of genetic imbalance of Th1 and Th2 genes and proteins in APS-treated type 1 DM NOD mice. Their studies observed about 5.47% changes in gene expression, of which 17 genes were of functional relation to immunity [51]. Further studies showed that APS demonstrates immunomodulatory effects on Th1 and Th2 cytokines. It was reported to have downregulated the expression levels of Th1 cytokines such as IL-12, TNF-*α*, and IFN*γ* and enhanced Th2 cytokines such as IL-4, IL-5, IL-6, and IL-10 [42, 45, 52]. APS also demonstrated a significant lowering effect on Th1/Th2 ratio [44, 53], an important apoptotic index that measures relatively lowered levels of Th1 per Th2 cytokines as an indication for reduced intracellular autoimmunity and inflammatory response [54]. The effect of APS on other inflammatory markers such as peroxisome-proliferator-activated receptor gamma (PPAR-*γ*), superoxide dismutase (SOD), and nitric oxide (NO) has also been studied. APS significantly enhanced the gene expression of PPAR-*γ* in a time- and dose-dependent manner [53] and promoted SOD anti-oxidation in type 1 DM models [42, 55]. It also lowered the expression of inducible nitric oxide synthase (iNOS) [42, 55]. PPAR-*γ*, NO, iNOS, and SOD among a variety of functions also play various roles in the stimulation and regulation of inflammatory response [56].

The effect of astragalin, a flavonoid isolate of AM, on apoptotic cytokines has also been studied. It showed an inhibitory effect on the production levels of TNF-*α*, IL-1, and IL-6 [57]. It was reported to have repressed the expression of these Th1 cells via NF-*κ*B inhibition. It has also been shown as exhibiting inhibitory effects on proinflammatory mediators similar to quercetin. It was shown to have attenuated the production of nitric oxide (NO) and repressed the expression and production levels of iNOS and cyclooxygenase-2 (COX-2) in J774A.1 mice macrophages [57, 58].

#### 4.1.2. Promotion of Antiapoptotic Response

APS has exhibited the potential to regulate a number of apoptosis-related proteins and enzymes. It demonstrated significant inhibitory effect on caspase-3 enzyme [45, 59] while enhancing the expression of B-cell lymphoma-2 (Bcl-2) [55] in type 1 DM models. Caspase-3 is noted for apoptosis execution, whereas Bcl-2 has apoptosis regulatory effects. APS was also positively correlated to increased galectin-1 levels in the muscles of type 1 DM mice. Its correlation with galectin-1 was further shown to have a negative regulatory effect on CD8^+^ T cells, an apoptosis-enhancing T cell [60]. APS has also been reported to have lowered the expression of Fas [42, 61, 62]. Fas is a member of the TNF family of receptors that expresses on cells to trigger their apoptosis. 

Formononetin, an O-methylated isoflavone, has been reported as inhibiting the activity of caspase-3. It was shown to have reduced caspase-3 levels in INS-1 cells [63]. It also lowered *in vitro* nitric oxide production and apoptotic signaling via a demonstrated inhibition of IL-1*β* and reduction of Bax/Bcl-2 ratio. It was also shown to have inhibited the activation of nuclear factor-kappaB (NF-*κ*B) [63].

### 4.2. Type 2 Diabetes Mellitus

Type 2 of DM is caused by insulin resistance and deficient glucose metabolism. All of the major constituents of AM have been shown to differentially lower high blood glucose levels and body weight and improve impaired glucose tolerance in type 2 diabetic models [64–67]. The postulated pharmacological processes include various glucose transportation and insulin signaling pathways that lead to insulin sensitivity and restoration of the proliferative ability of the pancreatic beta cells. 

#### 4.2.1. Promotion of Intracellular Glucose Transportation

The polysaccharides fraction has exhibited potentials of reducing hyperglycemia through the induction of glucose translocation enzymes and proteins. It has been studied as a promoter of increased glucose transporter protein-4 (GLUT4) levels. In a molecular expression study of the effect of APS on GLUT4, APS increased the expression and translocation of GLUT4 in skeletal muscle and adipose tissues [64, 68]. The GLUT4 is an insulin-regulated intracellular transporter noted for the mediation of glucose translocation into muscle and fat cells. Liu et al. (2010) analyzed the effect of APS on the GLUT4/protein kinase B (PKB) glucose transportation pathway in the skeletal muscles of insulin-resistant KKAy mice. APS was reported to have partially restored lowered activation levels of PKB and GLUT4 translocation [64]. 

#### 4.2.2. Regulation of Glucose and Lipid Metabolism

 Increased levels of circulating glucose, free fatty acids, and accumulation lipids in nonadipose tissues have been implicated in the development of insulin resistance and type 2 DM [69]. APS, ASS, and ASF have all shown differential regulatory effects on several glucose- and lipid-metabolizing enzymes, proteins, and receptors. The polysaccharides fraction has been the most widely studied. It has been shown to have enhanced the phosphorylation and activation of hepatic glycogen synthase and regulated the expression and activation of adenosine monophosphate-alpha (AMP-*α*) and acetyl-CoA carboxylase to alleviate glucose accumulation in *in vitro* skeletal muscle cells and KKAy mice models [65]. It also exhibited an upregulatory effect on the levels of adiponectin [70] and its receptor, adipo-R1 [71], in type 2 DM rats. It promoted the expression and activation of adenosine monophosphate protein kinase (AMPK) and its alpha-subunit, AMPK-alpha [65, 71, 72]. Adiponectin and AMPK are important activating factors for glucose and lipid metabolism in the liver, muscles, and adipocytes. Increased levels of their activity have been associated with reduced risk for type 2 DM [73, 74]. Other studies have demonstrated APS as regulating glucose and lipid metabolism through the promotion of peroxisome proliferator-activated receptor- (PPAR-) alpha activity and inhibition of the autonomic neurotransmitter neuropeptide-Y (NPY). The PPARs are a family of ligand-dependent transcription factors that control energy homeostasis through the regulation of carbohydrate and lipid metabolism. PPAR-alpha potentiates fatty acid catabolism and reduces circulating lipids [75]. APS enhanced the gene and protein expression of PPAR-*α* and improved the lipoprotein profiles of streptozotocin-induced diabetic hamsters [76]. Neuropeptide-Y is an autonomic neurotransmitter that induces increased food intake leading to obesity and type 2 DM. Chen et al. (2011) reported lowered levels of increased blood glucose and body weight in relation to neuropeptide-Y in streptozotocin-induced diabetic rats. APS was reported to have reduced the mRNA expression levels of neuropeptide-Y and its receptor neuropeptide-Y2 protein [77]. The effect of APS on aldose reductase, a glucose-metabolizing enzyme target implicated in high-glucose-induced diabetes complications [78], has also been studied. APS had no significant inhibitory effect on aldose reductase [79]. 

The saponins (ASS) and flavonoids (ASF) fractions exhibited their antagonizing effects on ascending blood glucose levels in type 2 DM rats through a common adiponectin and AMPK-metabolizing pathway. They increase the genetic and cellular expression of AMPK, adiponectin, and adipo-R1 levels in the liver and skeletal muscle of diabetic rats [70, 71]. The expression levels of AMPK and adipo-R1 induced by the saponins were reported to be more pronounced in the skeletal muscles than in the liver, whereas the flavonoids showed an increased effect in the liver than in the skeletal muscle [71]. 

Several *Astragalus *saponins isolates have been studied. Astragaloside II and isoastragaloside I exhibited regulatory effects on adiponectin and AMPK action. They significantly increased adiponectin levels and promoted the activation of AMPK in type 2 DM mice. Their induction of increased adiponectin levels was reported to be independent of PPAR*γ*, an adiponectin agonist [75, 80]. The *Astragalus* saponins astragalosides I and IV have demonstrated inhibitory effect on aldose reductase. They downregulated its activation levels to ameliorate accumulation of advanced glycation endproducts in both erythrocytes and nerve cells of diabetic rats [81]. 

The comparative effects of formononetin and calycosin isoflavonoids on the peroxisome-proliferator-activated receptors activation system have also been studied. Formononetin was reported to be more potent activator of PPAR*γ*-induced differentiation of 3T3-L1 preadipocyte than calycosin [18]. PPAR*γ* plays crucial role in the differentiation and maturity of fat cells [75].

#### 4.2.3. Alleviation of ER Stress and Induction of Insulin Signal Transduction

Stress responses in the endoplasmic reticulum (ER) have been associated with increased *β*-cell apoptosis rates, reduced beta cell mass, lowered insulin production, and increased insulin resistance in type 2 DM patients. APS has been reported as a negative regulator of key ER stress indicators such as phosphorylated protein kinase-like endoplasmic reticulum kinase (PERK), activating transcription factor-6 (ATF-6), glycogen synthase kinase 3 beta (GSK3*β*), and XhoI site-binding protein 1 (XBP1) in type 2 diabetes models. It relieved ER stress in type 2 DM SD rats through a significant decrease in the expression of PERK and inhibition of ATF-6 activity [82]. It also reduced the levels of the transcription repressor protein XBP1 and GSK3*β* in KKAy mice [83]. The inhibitory effect of APS on ATF-6 was further studied in relation to protein tyrosine phosphatase-1-B (PTP1B), a negative regulator of insulin-receptor signal transduction. ATF-6 inhibition was positively correlated with lowered expression and activation levels of PTP1B in experimental animals [67, 84, 85]. APS may have indirectly promoted insulin signaling via ER stress alleviation. Other insulin signaling studies have reported the upregulatory effect of APS on insulin receptors. APS was shown to have increased the levels of insulin receptor substrate-1 (IRS-1) and its beta transmembrane receptor (IR-*β*) subunit in muscle cells [84]. IRS-1 s key role in insulin signal transduction. Lowered levels of IRS-1 have been associated with increased susceptibility to type 2 DM [86, 87]. APS has also demonstrated regulatory effects on resistin, an insulin-resistance protein [88, 89]. It decreased the mRNA and protein expression levels of resistin in type 2 DM Wistar rats.

## 5. Pharmacological Prospects and Concluding Remarks

The anti-diabetic potential of *Astragalus membranaceus* has been progressively studied in the recent past. Its crude extracts have been reported in several ethnopharmacological studies as potential prospect for further anti-diabetic studies. Recent studies have analyzed its phytochemical constituents in elucidating its pharmacological significance to types 1 and 2 DM. Its polysaccharides, saponins, and flavonoids fractions and several isolated compounds have been studied. They all exhibited differential potentials of correcting the characteristic defects of inadequate insulin production, secretion, and action on target cells. The total polysaccharides fraction demonstrates significant activity to type 1 DM. It protects pancreatic beta cells from intracellular (autoimmune) cell death via the immunomodulation of several inflammatory and apoptotic cytokines, enzymes, and proteins. It demonstrated the potential to modulate T helper cells 1 and 2, reduce inflammatory response, and promote antioxidant activities towards antiapoptotic protection of pancreatic beta cells. Astragalin and formononetin also demonstrated regulatory effects on various inflammatory and apoptotic indicators.

The polysaccharides, saponins, and flavonoids fractions all exhibited significant activities to type 2 DM. They generally induce their hypoglycemic effects through various insulin sensitizing pathways. They all demonstrated regulatory effects on AMPK and adiponectin and its receptor adipo-R1. Astragaloside II and isoastragaloside I isolates were also associated with this effect. The polysaccharides fraction has been most extensively studied in relation to type 2 DM. It promotes insulin sensitization through various coordinated pathways towards intracellular glucose transportation, insulin signal transduction, and protection of pancreatic beta cells from apoptotic death. It promoted the PKB/Akt and -PPAR-*α* and -*γ* systems, activated insulin receptors, and regulated ER stress-related proteins and enzymes. The PKB/Akt system differentially coordinates PKB to glycogen synthase kinase 3 (GSK3), GLUT4, apoptotic caspases, IR, and IRS-1, among others, to induce glucose transportation and cell proliferation. The phosphorylation and activation of PKB lead to increased IRS-1 and GLUT4 activity for glucose translocation and insulin signaling. Its activation also results in the inactivation of GSK3 and caspase proteases to inhibit apoptosis [90, 91]. Stress-induced apoptosis in the endoplasmic reticulum of pancreatic and liver cells has also been related to reduced insulin production and increased insulin resistance [92, 93]. APS notably exhibited a negatively regulatory effect on PERK, ATF-6, and XBP1 ER stress indicators. The PPAR are a family of ligand-dependent transcription factors that control energy homeostasis through the regulation of carbohydrate and lipid metabolism [94]. They are also involved in the regulation of inflammatory responses [56, 75]. PPAR-alpha [75] and PPAR-gamma [94] have been associated with the regulation of DM. APS demonstrated activity to both of them to alleviate high blood glucose levels. The demonstrated regulatory effects of APS on these systems suggest its importance and prospects for further research and development for diabetes therapy.

Further studies on more single-compound isolates are important to understand the overall mechanisms and processes of anti-diabetic effects as well as their structure-activity relationships.

## References

[1] Wild S, Roglic G, Green A, Sicree R, King H (2004). Global prevalence of diabetes: estimates for the year 2000 and projections for 2030. *Diabetes Care*.

[2] Einhorn D (2004). Advances in diabetes for the millennium: insulin treatment and glucose monitoring CME. *MedGenMed Medscape General Medicine*.

[3] Rendell M (2004). Advances in diabetes for the millennium: drug therapy of type 2 diabetes. *Medscape General Medicine*.

[4] Lambert P, Bingley PJ (2002). What is type 1 diabetes?. *Medicine*.

[5] Berman BM, Swyers JP, Kaczmarczyk J (1999). Complementary and alternative medicine: herbal therapies for diabetes. *Journal of the Association for Academic Minority Physicians*.

[6] Jia W, Gao W, Xiao P (2003). Antidiabetic drugs of plant origin used in China: compositions, pharmacology, and hypoglycemic mechanisms. *Zhongguo Zhongyao Zazhi*.

[7] Wei DX, Yu NZ, Ya OZ (2011). Traditional Chinese medicines in treatment of patients with type 2 diabetes mellitus. *Evidence-Based Complementary and Alternative Medicine*.

[8] Li WL, Zheng HC, Bukuru J, De Kimpe N (2004). Natural medicines used in the traditional Chinese medical system for therapy of diabetes mellitus. *Journal of Ethnopharmacology*.

[9] Zang SF, Ning L, Ni HX, Zhang QL, Chen ZJ (2006). The effect of Astragalus on PPAR-*γ* mRNA expression in macrophage with Type2 Diabetic Mellitus. *Chinese Archives of Traditional Chinese Medicine*.

[10] Liang XC, Cui LY, Guo SS (2001). Clinical study of Jinmaitong composita on diabetic peripheral neuropathy. *Chinese Journal of Integrative Medicine*.

[11] Seely D, Mills E, Rachlis B (2006). Patients with diabetes using alternative medicine. *Contemporary Endocrinology (Evidence-Based Endocrinology)*.

[12] Keji C (1981). Understanding and treatment of diabetes mellitus by traditional Chinese medicine. *American Journal of Chinese Medicine*.

[13] Sang Z, Zhou L, Fan X, McCrimmon RJ (2010). Radixz astragali (Huangqi) as a treatment for defective hypoglycemia counterregulation in diabetes. *American Journal of Chinese Medicine*.

[14] Zang SF, Ning L, Ni HX, Zhang QL, Chen ZJ (2011). The effect of Astragalus on PPAR-*γ* mRNA expression in macrophage with type2 diabetic mellitus. *Chinese Archives of Traditional Chinese Medicine*.

[15] Chao M, Zou D, Zhang Y (2009). Improving insulin resistance with traditional Chinese medicine in type 2 diabetic patients. *Endocrine*.

[16] Zhang DQ, Zhang JJ, Wang JX (2005). Effects of Qilan Tangzhining capsule on glucose and lipid metabolism in rats with diabetes mellitus and hyperlipemia. *Zhongguo Zhongyao Zazhi*.

[17] Qin QJ, Niu JY, Wang ZX, Xu WJ, Qiao ZD, Gu Y (2012). *Astragalus membranaceus* Inhibits Inflammation via Phospho-P38 Mitogen-Activated Protein Kinase (MAPK) and Nuclear Factor (NF)-*κ*B Pathways in Advanced Glycation End Product-Stimulated Macrophages. *International Journal of Molecular Sciences*.

[18] Shen P, Liu MH, Ng TY, Chan YH, Yong EL (2006). Differential effects of isoflavones, from *Astragalus membranaceus* and Pueraria Thomsonii, on the activation of PPAR*α*, PPAR*γ*, and adipocyte differentiation in vitro. *Journal of Nutrition*.

[19] WHO (1999). *Radix Astragali*.

[20] Thorne Research Incoporated (2003). Astragulus membranaceus monograph alternative medicine review. *Thorne Research Incoporated*.

[21] Liu X, Wang M, Wu H, Zhao X, Li H (1994). Isolation of astragalan and its immunological activities. *Tianran Chanwu Yanjiu Yu Kaifa*.

[22] Fang SD, Chen Y, Ye CQ, Zhai SK, Shen ML (1982). Studies of the active principles of Astragalus Mongholicus Bunge I. isolation, characterization and biological effect of its polysaccharides. *Chinese Journal of Organic Chemistry*.

[23] Zou YY, Gu XQ, Chen Q (1987). Investigation of the polyphase liposomal bipolysaccharides-Part 1. Selection and analysis of the effective ingredients in the two polysaccharides. *Journal of Shenyang Pharmaceutical University*.

[24] Tomoda M, Shimizu N, Ohara N, Gonda R, Ishii S, Otsuki H (1991). A reticuloendothelial system-activating glycan from the roots of *Astragalus membranaceus*. *Phytochemistry*.

[25] Mu LX, Zhu L, Zhao AH, Zhou MM, Jia W (2009). Study on extraction and purification of polysaccharides from *Astragalus membranaceus*. *Zhong Yao Cai*.

[26] Wang SC, Shan JJ, Wang ZT, Hu ZB (2006). Isolation and structural analysis of an acidic polysaccharide from *Astragalus membranaceus* (Fisch.) Bunge. *Journal of Integrative Plant Biology*.

[27] Jing L, Zhen ZZ, Biao CH (2011). Review of Astragali radix. *Chinese Herbal Medicines*.

[28] Kajimura K, Takagi Y, Ueba N (1996). Protective effect of Astragali Radix by intraperitoneal injection against Japanese encephalitis virus infection in mice. *Biological and Pharmaceutical Bulletin*.

[29] Bombardelli E, Pozzi R Polysaccharides with immunomodulating properties from *Astragalus membranaceus* and pharmaceutical compositions containing them. http://brevets-patents.ic.gc.ca/opic-cipo/cpd/eng/patent/2035948/summary.html.

[30] Kitagawa I, Wang HK, Saito M (1983). Saponin and Sapogenol. XXXV. Chemical constituents of Astragali Radix, the root of *Astragalus membranaceus* Bunge. (2). Astragalosides I, II, and IV, acetylastragaloside I and isoastragalosides I and II. *Chemical and Pharmaceutical Bulletin*.

[31] Kitagawa I, Wang HK, Saito M, Yoshikawa M (1983). Saponin and Sapogenol. XXXVI. Chemical constituents of Astragali Radix, the root of *Astragalus membranaceus* Bunge. (3). Astragalosides III, V, and VI. *Chemical and Pharmaceutical Bulletin*.

[32] Kitagawa I, Wang HK, Yoshikawa M (1983). Saponin and Sapogenol. XXXVII. Chemical constituents of Astragali Radix, the root of *Astragalus membranaceus* Bunge. (4) Astragalosides VII and VIII. *Chemical and Pharmaceutical Bulletin*.

[33] Kim JS, Yean M, Lee E (2008). Two new cycloartane saponins from the roots of *Astragalus membranaceus*. *Chemical and Pharmaceutical Bulletin*.

[34] Chu C, Cai H, Ren M (2010). Characterization of novel astragaloside malonates from Radix Astragali by HPLC with ESI quadrupole TOF MS. *Journal of Separation Science*.

[35] He ZQ, Findlay JA (1991). Constituents of *Astragalus membranaceus*. *Journal of Natural Products*.

[36] Xu Q, Ma XQ, Liang XM (2007). Determination of astragalosides in the roots of Astragalus spp. using liquid chromatography tandem atmospheric pressure chemical ionization mass spectrometry. *Phytochemical Analysis*.

[37] Lee EJ, Yean MH, Jung HS, Kim JS, Kang SS (2008). Phytochemical studies on Astragalus root (2)-flavonoids and a lignan. *Natural Product Sciences*.

[38] Zhang LJ, Liu HK, Hsiao PC (2011). New isoflavonoid glycosides and related constituents from astragali radix (*Astragalus membranaceus*) and their inhibitory activity on nitric oxide production. *Journal of Agricultural and Food Chemistry*.

[39] Lin LZ, He XG, Lindenmaier M (2000). Liquid chromatography-electrospray ionization mass spectrometry study of the flavonoids of the roots of Astragalus mongholicus and A. membranaceus. *Journal of Chromatography A*.

[40] Wu XY, Chen W, Yu MH (2008). Effect of astragalus polysaccharides on expression of IL-4 and IFN-*γ* in non-obese diabetic mice. *Chinese Journal of Rehabilitation Theory and Practice*.

[41] Chen W, Liu F, Yu M, Zhu Q, Zhu X (2001). Astragalus polysaccharide prevent type 1 diabetes in nonobese diabetic mice. *Fudan University Journal of Medical Sciences*.

[42] Chen W, Li Y-M, Yu M-H (2008). Astragalus polysaccharides: an effective treatment for diabetes prevention in NOD mice. *Experimental and Clinical Endocrinology and Diabetes*.

[43] Chen W, Xia YP, Yu MH, Shi XM (2007). Astragalus polysaccharides effect on pancreatic histopathology. *China Journal of Modern Medicine*.

[44] Li RJ, Qiu SD, Chen HX, Tian H, Wang HX (2007). The immunotherapeutic effects of Astragalus polysaccharide in type 1 diabetic mice. *Biological and Pharmaceutical Bulletin*.

[45] Li RJ, Qiu SD, Chen HX, Tian H, Liu G (2007). Effect of Astragalus polysaccharide on pancreatic cell mass in type 1 diabetic mice. *Zhongguo Zhongyao Zazhi*.

[46] Chen W, Li YM, Yu MH, Shi XM (2007). Immunoloregulation effects of Astragalus Polysaccharides on T helper lymphocyte subgroups in nonobese diabetic Mice. *China Journal of Modern Medicine*.

[47] Wong FS, Siew LK, Scott G (2009). Activation of insulin-reactive cd8 t-cells for development of autoimmune diabetes. *Diabetes*.

[48] Harty JT, Tvinnereim AR, White DW (2000). Cd8+ T cell effector mechanisms in resistance to infection. *Annual Review of Immunology*.

[49] Dong C, Flavell RA (2000). Cell fate decision: T-helper 1 and subsets in immune responses. *Arthritis Research*.

[50] Crane IJ, Forrester JV (2005). Th1 and Th2 lymphocytes in autoimmune disease. *Critical Reviews in Immunology*.

[51] Chen W, Yu MH (2004). Effects of Astragalus polysaccharide on gene expression profiles in islets of NOD mice with microarray technique Chinese. *Chinese Journal of Endocrinology and Metabolism*.

[52] Chen W, Li YM, Yu MH (2007). Astragalus polysaccharides influence ultrastructure of islets and Th1/Th2 cvtokine gene expression of pancrease in NOD mice. *Chinese Journal of Endocrinology and Metabolism*.

[53] Li RJ, Qiu SD, Chen HX, Wang LR (2008). Immunomodulatory effects of Astragalus polysaccharide in diabetic mice. *Zhong Xi Yi Jie He Xue Bao*.

[54] Azar ST, Tamim H, Beyhum HN, Zouhair Habbal M, Almawi WY (1999). Type I (insulin-dependent) diabetes is a Th1- and Th2-mediated autoimmune disease. *Clinical and Diagnostic Laboratory Immunology*.

[55] Chen W, Yu MH, Li YM (2007). Effects of astragalus polysaccharides on ultrastructure and oxidation/ apoptosis related cytokines’ gene expression of non-obese diabetic mice’s islets. *Fudan University Journal of Medical Sciences*.

[56] Guri AJ, Mohapatra SK, Horne WT, Hontecillas R, Bassaganya-Riera J (2010). The Role of T cell PPAR *γ* in mice with experimental inflammatory bowel disease. *BMC Gastroenterology*.

[57] Soromou LW, Chen N, Jiang L (2012). Astragalin attenuates lipopolysaccharide-induced inflammatory responses by down-regulating NF-*κ*B signaling pathway. *Biochemical and Biophysical Research Communications*.

[58] Kim MS, Kim SH (2011). Inhibitory effect of astragalin on expression of lipopolysaccharideinduced inflammatory mediators through NF-*κ*B in macrophages. *Archives of Pharmacal Research*.

[59] Mao SM, Li CD, Wang L, Wang J, Dai G, Kang B (2009). Effect and mechanism of astragalus polysaccharides on apoptosis of the islet beta cell in diabetes mellitus rats. *Chinese Pharmacological Bulletin*.

[60] Zhou XJ, Xu YC, Yang GM, Li F (2011). Increased galectin-1 expression in muscle of Astragalus polysaccharide-treated Type 1 diabetic mice. *Journal of Natural Medicines*.

[61] Li CD, Li JJ, Wang L (2011). Inhibitory effect of astragalus polysaccharides on apoptosis of pancreatic beta-cells mediated by Fas in diabetes mellitus rats. *Zhong Yao Cai*.

[62] Mao SM, Li CD, Wang L (2010). Effects of astragalus polysaccharides on the ultrastructure and Fas expression of pancreatic beta-cells in diabetes mellitus rats. *Chinese Pharmacological Bulletin*.

[63] Wang Y, Zhu Y, Gao L (2012). Formononetin attenuates IL-1*β*-induced apoptosis and NF-*κ*B activation in INS-1 cells. *Molecules*.

[64] Liu M, Wu K, Mao X, Wu Y, Ouyang J (2010). Astragalus polysaccharide improves insulin sensitivity in KKAy mice: regulation of PKB/GLUT4 signaling in skeletal muscle. *Journal of Ethnopharmacology*.

[65] Zou F, Mao XQ, Wang N, Liu J, Ou-Yang JP (2009). Astragalus polysaccharides alleviates glucose toxicity and restores glucose homeostasis in diabetic states via activation of AMPK. *Acta Pharmacologica Sinica*.

[66] Liao WP, Shi YG (2007). Effect of astragalus polysaccharides and soy isoflavones on glucose metabolism in diabetic rats. *Acta Academiae Medicinae Militaris Tertiae*.

[67] Wang N, Zhang D, Mao X, Zou F, Jin H, Ouyang J (2009). Astragalus polysaccharides decreased the expression of PTP1B through relieving ER stress induced activation of ATF6 in a rat model of type 2 diabetes. *Molecular and Cellular Endocrinology*.

[68] Liu HF, Ren YH, Han ZX (2011). Effect of astragalus poly saccharides on insulin resistance and gene expression of GLUT4 in type 2 diabetes mellitus rats. *Chinese Journal of Gerontology*.

[69] Kelley DE, Goodpaster BH (2001). Skeletal muscle triglyceride: an aspect of regional adiposity and insulin resistance. *Diabetes Care*.

[70] Li N, Fan Y, Jia XM, Ma Z, Lin SR (2011). Effect of astragali radix active ingredients on serum insulin and adiponectin in diabetes rats. *Chinese Journal of Experimental Traditional Medical Formulae*.

[71] Li N, Fan Y, Jia XM, Lin SR, Ma Z (2011). Effect of Astragalus active ingredients on AdipoR1 and AMPK mRNA expression in diabetic rats. *China Journal of Traditional Chinese Medicine and Pharmacy*.

[72] Wu DH, Wang FJ, Deng J (2009). Effect of APS on the expression of phosphyorylation of AMPK in liver tissue of type 2 diabetic rat. *Chinese Journal of Microcirculation*.

[73] Spranger J, Kroke A, Möhlig M (2003). Adiponectin and protection against type 2 diabetes mellitus. *The Lancet*.

[74] Lele RD (2010). Pro-insulin, C peptide, glucagon, adiponectin, TNF*α*, AMPK: neglected players in type 2 diabetes mellitus. *Journal of Association of Physicians of India*.

[75] Koh EH, Kim M, Park J (2003). Peroxisome proliferator-activated receptor (PPAR)-*α* activation prevents diabetes in OLETF rats: comparison with PPAR-*γ* activation. *Diabetes*.

[76] Wei C, Yanping X, Xuelan Z (2012). The critical role of Astragalus polysaccharides for the improvement of PPRA*α*-mediated lipotoxicity in diabetic cardiomyopathy. *PLOS One*.

[77] Chen YS, Liu HQ, Qin W, Luo XM (2011). Effect of Astragalus polysaccharides on neuropeptide Y and its receptor Y_2_ expression in streptozotocin-induced diabetic rats. *Journal of Liaoning University of Traditional Chinese Medicine*.

[78] Narayanan S (1993). Aldose reductase and its inhibition in the control of diabetic complications. *Annals of Clinical and Laboratory Science*.

[79] Yang HZ, Zhou MM, Zhao AH, Xing SN, Fan ZQ, Jia W (2009). Study on effects of baicalin, berberine and Astragalus polysaccharides and their combinative effects on aldose reductase in vitro. *Zhong Yao Cai*.

[80] Xu A, Wang H, Hoo RLC (2009). Selective elevation of adiponectin production by the natural compounds derived from a medicinal herb alleviates insulin resistance and glucose intolerance in obese mice. *Endocrinology*.

[81] Yu J, Zhang Y, Sun S (2006). Inhibitory effects of astragaloside IV on diabetic peripheral neuropathy in rats. *Canadian Journal of Physiology and Pharmacology*.

[82] Wang N, Mao XQ, Wang S, Zhang C, Zou F, Ouyang JP (2007). Effects of APS on reducing ER stress and increasing insulin sensitivity in a rat model of type 2 diabetes. *Journal of Public Health and Preventive Medicine*.

[83] Mao XQ, Wu Y, Wu K (2007). Astragalus polysaccharide reduces hepatic endoplasmic reticulum stress and restores glucose homeostasis in a diabetic KKAy mouse model. *Acta Pharmacologica Sinica*.

[84] Wu Y, Ou-Yang JP, Wu K, Wang Y, Zhou Y, Wen C (2005). Hypoglycemic effect of Astragalus polysaccharide and its effect on PTP1B. *Acta Pharmacologica Sinica*.

[85] Mao X, Yu F, Wang N (2009). Hypoglycemic effect of polysaccharide enriched extract of *Astragalus membranaceus* in diet induced insulin resistant C57BL/6J mice and its potential mechanism. *Phytomedicine*.

[86] Kovacs P, Hanson RL, Lee Y (2003). The role of insulin receptor substrate-1 gene (IRS1) in type 2 diabetes in Pima Indians. *Diabetes*.

[87] Zeggini E, Parkinson J, Halford S (2004). Association studies of insulin receptor substrate 1 gene (IRS1) variants in type 2 diabetes samples enriched for family history and early age of onset. *Diabetes*.

[88] Liu HF, Zhang Y, Hu Z (2011). Effect of Astragalus polysaccharides on gene expression of resistin in type 2 diabetes mellitus rats. *Journal of Mudanjiang Medical University*.

[89] Liu HF, Chen HJ, Wang GY, Han ZX, Zhang J (2012). Effect of astragalus polysaccharides on insulin resistance and protein pxpression of resistin in type 2 diabetes mellitus rats. *Food and Nutrition in China*.

[90] Lawlor MA, Alessi DR (2001). PKB/Akt: a key mediator of cell proliferation, survival and insulin responses?. *Journal of Cell Science*.

[91] Karlsson HKR, Zierath JR, Kane S, Krook A, Lienhard GE, Wallberg-Henriksson H (2005). Insulin-stimulated phosphorylation of the Akt substrate AS160 is impaired in skeletal muscle of type 2 diabetic subjects. *Diabetes*.

[92] Özcan U, Cao Q, Yilmaz E (2004). Endoplasmic reticulum stress links obesity, insulin action, and type 2 diabetes. *Science*.

[93] Fu Z, Gilbert ER, Liu DM (2013). Regulation of insulin synthesis and secretion and pancreatic beta-cell dysfunction in diabetes. *Current Diabetes Reviews*.

[94] Reginato MJ, Lazar MA (1999). Mechanisms by which thiazolidinediones enhance insulin action. *Trends in Endocrinology and Metabolism*.

